# Electronic Spectroscopy of Phthalocyanine and Porphyrin Derivatives in Superfluid Helium Nanodroplets

**DOI:** 10.3390/molecules22081244

**Published:** 2017-07-25

**Authors:** Alkwin Slenczka

**Affiliations:** Institute for Physical and Theoretical Chemistry, University of Regensburg, 93053 Regensburg, Germany; alkwin.slenczka@chemie.uni-regensburg.de; Tel.: +49-941-943-4483

**Keywords:** phthalocyanine, porphyrin, helium droplets, electronic spectroscopy, microsolvation

## Abstract

Phthalocyanine and porphyrin were among the first organic compounds investigated by means of electronic spectroscopy in superfluid helium nanodroplets. Superfluid helium nanodroplets serve as a very gentle host system for preparing cold and isolated molecules. The uniqueness of helium nanodroplets is with respect to the superfluid phase which warrants the vanishing viscosity and, thus, minimal perturbation of the dopant species at a temperature as low as 0.37 K. These are ideal conditions for the study of molecular spectra in order to analyze structures as well as dynamic processes. Besides the investigation of the dopant species itself, molecular spectroscopy in helium droplets provides information on the helium droplet and in particular on microsolvation. This article, as part of a special issue on phthalocyanines and porphyrins, reviews electronic spectroscopy of phthalocyanine and porphyrin compounds in superfluid helium nanodroplets. In addition to the wide variety of medical as well as technical and synthetical aspects, this article discusses electronic spectroscopy of phthalocyanines and porphyrins in helium droplets in order to learn about both the dopant and the helium environment.

## 1. Introduction

The physical properties of liquid helium, and in particular the superfluid phase, qualify as an ideal host system for preparing cold and isolated molecules [1,2,3,4,5,6]. Below 2.5 MPa of pressure, helium remains liquid down to the absolute zero point of temperature. Thereby, helium is unique in passing a phase transition at about 2.17 K from a fluid to a superfluid, exhibiting vanishing viscosity and superior heat conductivity. The major problem when making use of superfluid helium as a host system is the poor solubility of molecules. When dissolved into liquid helium, molecules tend to coagulate among each other and finally stick to the walls of the helium dewar. These problems have been overcome by generating a beam of superfluid helium droplets inside a vacuum machine in which each carries a single particle of the species of interest [1,2,3]. The droplet beam is generated by a low-temperature expansion of pressurized helium through a small orifice into a vacuum. Depending on the expansion conditions, either an expanding gas condenses to form droplets or a liquid expands and breaks up into droplets. It is evident that the expansion conditions, such as stagnation pressure (10–100 atm), nozzle temperature (4–20 K), and nozzle diameter (5–10 μm), determine the size distribution of the droplets in the beam. In fact, the droplet size can easily be tuned from 10${}^{3}$ to almost 10${}^{8}$ helium atoms or even beyond (cf. [2,3] and references therein). The well-known density of superfluid helium allows for estimating the diameter as well as the cross section of these droplets as 2.22 Å N${}^{1/3}$ and 15.5 Å${}^{2}$ N${}^{2/3}$, respectively, where N is the number of atoms forming the droplet [7]. As is well known, under low pressure conditions in a vacuum machine the boiling temperature is significantly reduced. As a consequence, evaporative cooling of the droplets continues far below the limit at standard pressure and warrants for a temperature of only 0.37(1) K as deduced from rotationally resolved infrared (IR) spectra of molecules in helium droplets [8,9]. Besides continuous flow droplet sources, solenoid-driven valves have been used to generate intense pulsed droplet sources [10,11,12,13]. However, the work described below has exclusively been obtained from continuous flow droplet sources. The dopant species cannot be premixed to the expanding helium for the following reason. At the given temperatures any substance other than helium condenses and, thereby clogs the cold nozzle immediately. Moreover, quantitative control of the number of dopant species per droplet might be rather difficult. Instead, the droplets are doped by the so-called pick-up procedure [1]. On the flight through a confined volume filled with a gas phase sample under a certain vapor pressure, the droplets capture individual particles by collisions. This pick-up procedure obeys Poisson statistics which allows for quantitative control of the number of particles doped into the droplets. In any case the temperature of the gas phase sample exceeds that of the droplets by far. Consequently, energy dissipates from the captured dopant to the helium droplet. The dissipation of energy reactivates evaporative cooling of the entire system. Within picoseconds (ps) after the pick-up the doped droplet returns to the same thermal conditions as before doping. The intensity patterns of rotationally resolved IR spectra of hundreds of molecules and molecular compounds are consistent in revealing a temperature of only 0.37(1) K for bosonic helium. This is safe below the lambda point of helium. In view of numerous other experimental observables there is no doubt about the superfluidity of these helium droplets [2,3,8,14].

Electronic spectroscopy of molecules suffers from a high density of vibrational and rotational states. Any effort to reduce the population distribution pays off when analyzing those spectra. The cooling of a molecular sample helps to concentrate the population distribution to a manageable number of quantum states. Despite the embedding sample particles isolated from each other into a solid rare gas matrix, supersonic expansion of a gas into vacuum is a widely applied technique for the preparation of cold isolated molecules. Helium droplet spectroscopy can be seen as a hybrid of both [1,2,3]. The latter technique is tuned up for the generation of helium nanodroplets while the doping with foreign particles relates to matrix isolation spectroscopy. Compared to supersonic jet expansion and matrix isolation, the doping into helium droplets is unbeatable with respect to efficient cooling and acceptable level of perturbation by the host environment. Besides accessing clearly the sub-Kelvin regime of temperature, the thermalization to the droplet temperature concerns all degrees of freedom with identical efficiency. Electronic, vibrational, rotational, and translational energies are cooled down to an equilibrium temperature of 0.37 K. Only spin-related populations such as for ortho and para of hydrogen molecules do not thermalize within the typical lifetime of a droplet experiment. Besides structural analysis of the dopant, the conditions in helium droplets are interesting for studying photo-induced dynamic processes of the dopant species. High thermal conductivity, high efficiency of energy dissipation, and vanishing viscosity allow insight into regions of the interaction potential which are otherwise unaccessible [6,15,16,17].

In most cases, intramolecular structure and intra- as well as inter-molecular dynamics are deduced from the frequencies of individual vibronic transitions identified in electronic spectra [6]. In addition to resonance frequencies, the line shape is an important source of information [17,18]. Line shape analysis profits immensely from the sub-Kelvin temperature range, especially in the case of dopant species as large as phthalocyanines and porphyrins. For molecular spectroscopy in superfluid helium nanodroplets the line shape bears information on microsolvation in superfluid helium. In the first place each electronic resonance is accompanied by a phonon wing (PW) representing excitations of the helium environment which are coupled to the molecular transition [18]. The bare molecular resonance is called zero phonon line (ZPL). However, the ZPL is not free of helium-induced signatures [17,19]. The line shape of both the PW and the ZPL carries information on the helium environment and on microsolvation in superfluid helium. Empirical interpretations of helium-induced spectroscopic signatures obtained in the line shape of ZPL and PW reveal the model of a solvation complex consisting of the dopant particle surrounded by a layer of localized helium atoms [20]. This model receives further empirical evidence straightforwardly from the dopant to helium van der Waals interaction which exceeds the helium-to-helium interaction in most cases by orders of magnitude. Increased moments of inertia observed in rotationally resolved spectra from molecules in helium droplets provide additional evidence from the experimental side [21].

Another interesting aspect of helium droplets is the design of molecular complexes. The pick-up technique allows for multiple doping of droplets with particles of identical and/or different species. The complexes generated by van der Waals attraction inside the helium droplet are readily cooled down to the droplet temperature of 0.37 K. Moreover, the transparency of superfluid helium over the entire spectral range of relevance for molecular spectroscopy allows for picking up radical species from a hot plasma source without radiative heating of the droplet. The final temperature of the complexes of only 0.37 K is unique compared to alternative techniques. Thus, numerous configurations of radical species have been decoded for the first time [9].

This article is focused on reviewing systematic studies on electronic spectroscopy of phthalocyanine and porphyrin derivatives in helium droplets. Thereby, the analysis of molecular properties as well as of details concerning microsolvation will be highlighted. Initially, an extensive investigation of electronic spectra of bare phthalocyanine in helium droplets will be presented, revealing both information on the dopant species as well as on the helium environment. Starting from this example, phthalocyanine derivatives and porphyrin derivatives are added to the discussion. While the experimental work discussed in the following deals exclusively with the classical method of fluorescence excitation, dispersed emission, and in one case with pump probe spectra, a vast development of high resolution spectroscopy in the frequency domain and the time domain has come up and is established in the files of molecules in helium droplets, which will not be subject of this article. Thus, this article is a spotlight on particular, mostly experimental work on helium droplet spectroscopy of phthalocyanines and porphyrins, which is written for the special issue on phthalocyanines and porphyrins. It aims to introduce organic chemists to the spectroscopic work on phthalocyanines and porphyrins from a physico-chemical perspective.

## 2. Phthalocyanine at 0.37 Kelvin inside a Helium Droplet

The fluorescence excitation spectrum in company with a dispersed emission spectrum is a firm fundament for the analysis of structure and dynamics of molecules. As discussed above, any molecular species embedded into a superfluid helium droplet is immediately (within ps) cooled down to a temperature of only 0.37 K. This is fortunate if not mandatory in particular for the interpretation of electronic spectra of larger molecules such as phthalocyanine and porphyrin derivatives. Under low temperature conditions and in particular in the sub-Kelvin regime, the analysis becomes much easier and more precise. A major part of this chapter will be devoted to reviewing the electronic spectroscopy of phthalocyanine in superfuid helium droplets. Besides tetracene [18,22,23,24,25,26,27,28,29,30] and glyoxal [18,28,31,32,33,34], phthalocyanine is the molecule with the most elaborate spectroscopic investigation by means of electronic spectroscopy in helium droplets [10,28,29,30,35,36,37,38,39]. In addition, we will cross-check the interpretations of the phthalocyanine spectra with data obtained for other phthalocyanine and porphyrin derivatives.

### 2.1. Vibronic Frequencies

**Figure 1:**
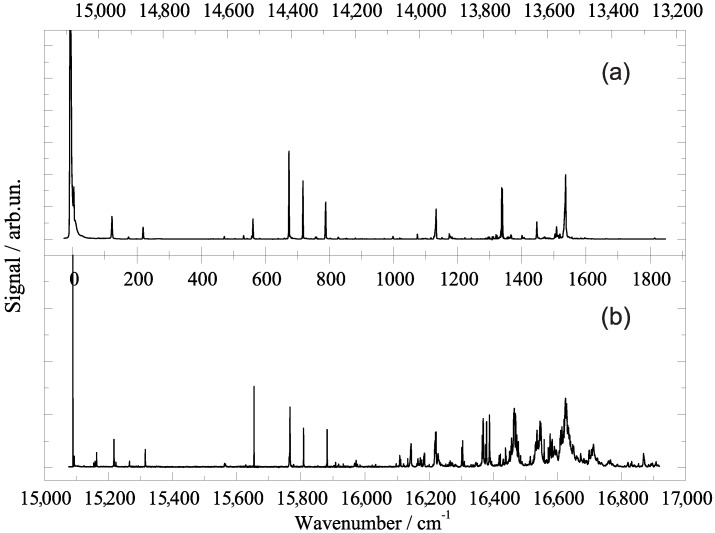
Electronic spectra of phthalocyanine in superfluid helium droplets at an average size of 20,000 atoms. (**a**) Dispersed emission spectrum recorded upon excitation at the electronic origin; (**b**) fluorescence excitation spectrum starting at the electronic origin of the S${}_{1}$–S${}_{0}$ transition. Top and bottom abscissa show the corresponding absolute wavenumber scale while the center abscissa is adjusted to the common origin of both spectra.

The presentation of experimental data starts with a comparative view at the fluorescence excitation spectrum (panel (b)) and the dispersed emission spectrum (panel (a)) of phthalocyanine in superfluid helium droplets as plotted in Figure 1. The fluorescence excitation spectrum starts at the low frequency side with a first and most intense resonance at 15,088.9 cm${}^{-1}$ which marks the electronic origin of phthalocyanine in superfluid helium droplets. Within the following 1800 cm${}^{-1}$ the spectrum of cold phthalocyanine reveals the vibrational fine structure of the S${}_{1}$ state of phthalocyanine which beyond 1000 cm${}^{-1}$ in excess to the electronic origin is superimposed by coupling of the otherwise forbidden S${}_{2}$ state. For the following discussion we will restrict ourselves to the S${}_{0}$–S${}_{1}$ transitions. On comparison with corresponding gas phase data, phthalocyanine exhibits a helium-induced solvent shift of about −42 cm${}^{-1}$ [18,21,28,40]. For vibrational frequencies the solvent shifts are in the order of 1 cm${}^{-1}$ (cf. Refs. [29,40,41,42,43]). Disregarding the perturbation by the second electronic state the line widths of vibronic transitions are all below 1 cm${}^{-1}$. The dispersed emission spectrum (panel (a)) recorded upon excitation at the electronic origin is plotted with an inverted frequency scale (top axis). The leading and most intense resonance in the emission at the high frequency side is found at 15,089 cm${}^{-1}$ [35,36,37] which is in perfect coincidence with the origin in excitation. This resonance is followed by a series of vibronic transitions which reveals the vibrational fine structure of the electronic ground state of phthalocyanine. Within the limits set by the spectral resolution, each of the resonances resolved in the dispersed emission spectrum coincides almost perfectly with a resonance in the fluorescence excitation spectrum. The resonances in the emission spectrum are also very sharp. However, the line shape is an apparatus function which hides the molecular line shape. Despite this limitation, the spectral resolution obtained in helium droplets exceeds what has been published in the gas phase by far [40,41,42,43].

The wealth of information summarized so far reveals many details on the dopant species as well as on its solvation in helium droplets. Starting with molecular properties we look at the left side of Figure 1. The coincidence of the origins in both spectra excludes photon-induced intramolecular dynamics upon excitation to the S${}_{1}$ state. Moreover, the coincidence of vibronic resonances in both spectra speaks for high similarity of binding conditions in both electronic states. Finally, the concentration of signal intensity at the electronic origin, the rather small intensity of vibronic transitions, and the missing of vibrational overtones speak for a pair of electronic states which are almost identical with respect to the structure and dynamic conditions. The obviously negligible structural changes upon excitation from S${}_{0}$ to S${}_{1}$ are due to the rigidity of phthalocyanine. This well-known and characteristic property of phthalocyanine and porphyrin derivatives is nicely confirmed by electronic spectra in superfluid helium droplets. The helium solvation shift of −42 cm${}^{-1}$ and the small line widths at the electronic origin and at vibronic transitions provide information on microsolvation of phthalocyanine in helium droplets. Sharp transitions speak for solvation inside the droplet. In contrast, severe line broadening is observed in electronic spectra of dopants residing on the surface of the droplet. The two cases are named as heliophilic or heliophobic, respectively. Heliophilic molecules are energetically stabilized upon solvation in liquid helium. A solvent shift to the red reveals a stronger stabilization of the S${}_{1}$ state than that of the S${}_{0}$ state whereas for a shift to the blue it is vice versa. While the solvent shift of electronic and vibrational frequencies differs by orders of magnitude, both values are found within a limit of ±1%. Empirically, this limit is a rule of thumb for helium-induced solvent shifts for electronic and vibrational frequencies. In particular, with respect to electronic frequencies helium is uniquely gentle. Moreover, a helium-induced shift of ≤±1% is almost the accuracy of theoretical transition frequencies. On the other hand, one can state that the helium environment exhibits a remarkable sensitivity on electronic excitation even though the S${}_{0}$ and the S${}_{1}$ states of phthalocyanine are very similar. Phthalocyanine in helium droplets reveals more about this sensitivity as to be discussed below.

**Figure 2:**
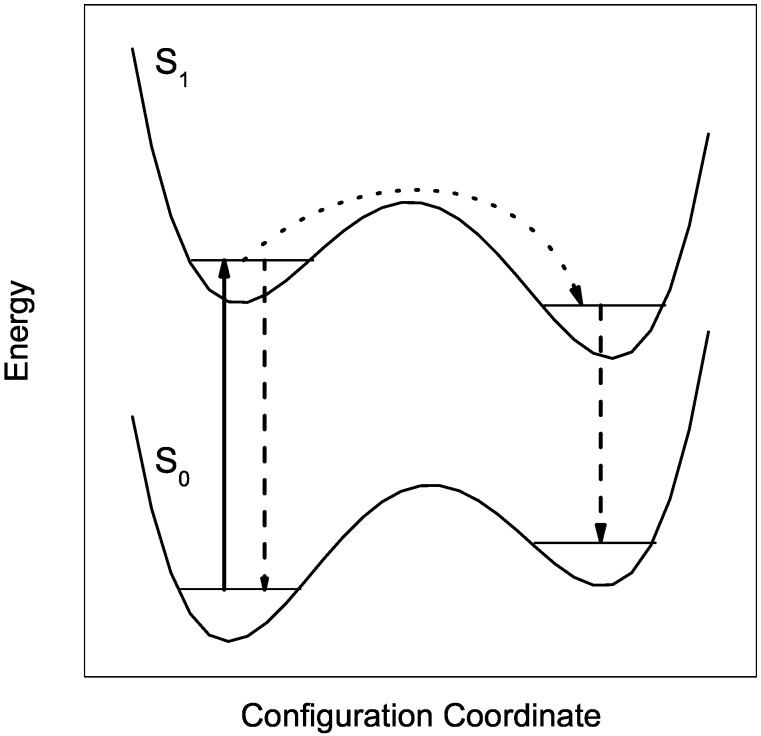
Model potential of the phthalocyanine solvation complex explaining dual emission (dashed vertical arrows) upon electronic excitation (full vertical arrow) by partial relaxation (dotted curved arrow) of the configuration of the solvation complex.

**Figure 3:**
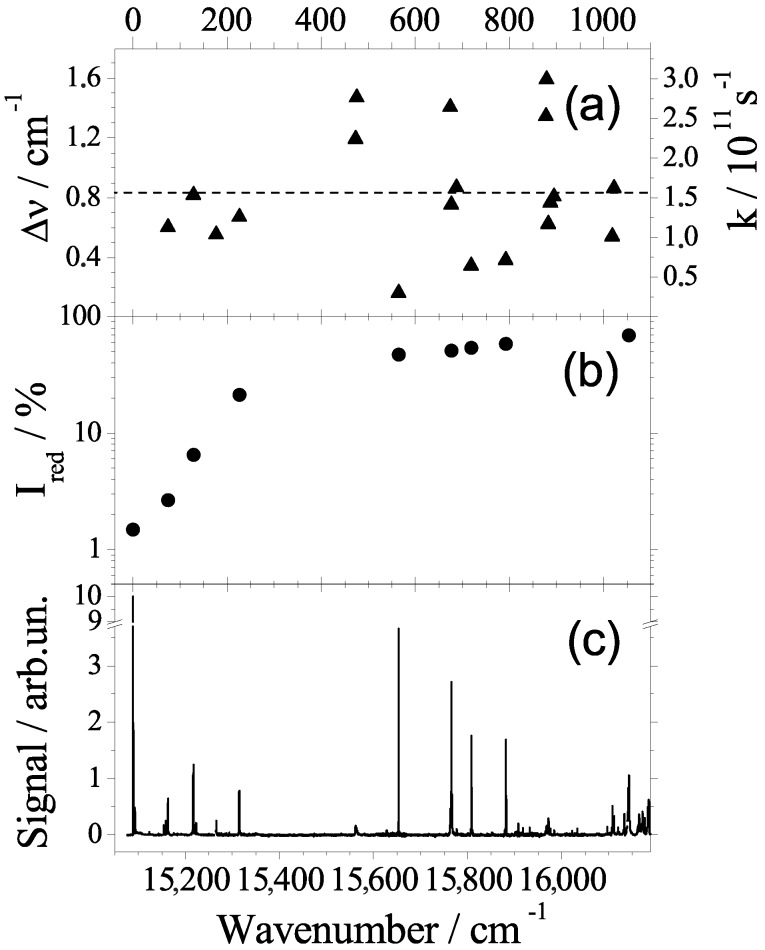
(**a**) Lorentzian full widths at half maximum (FWHM) abbreviated by $\Delta \bar{\nu }$ (left axis) and the corresponding decay rates k (right axis) of vibronic transitions of phthalocyanine in helium droplets. The horizontal line marks the average value; (**b**) Logarithmic plot of the relative contribution I${}_{r}ed$ in the decay path of electronically excited phthalocyanine to a relaxation of the solvation complex prior to radiative decay (for details, see the text). Panel (**a**) and (**b**) are plotted as function of the excitation energy (bottom abscissa) or vibrational excitation energy (top abscissa); Panel (**c**) visualizes the correlation of the data in (**a**) and (**b**) to the corresponding resonances in the fluorescence excitation spectrum of phthalocyanine in helium droplets.

Further important information has been gained from dispersed emission spectra upon excitation at vibronic levels [16,35,36,37]. With respect to intramolecular excitation energy, the helium environment serves as dissipative environment similar as known from solid matrices. In particular, any excitation energy in excess to the electronic origin dissipates into the helium environment prior to radiative decay. Thus, emission is only observed from the ground level of S${}_{1}$ a mechanism which is also well-known for matrix-isolated molecules. Moreover, the helium environment shows an interesting response to electronic excitation. Upon electronic excitation, the electron density distribution is changed even though the nuclear configuration of phthalocyanine remains almost unchanged. However, the helium environment sensitizes the change of the electron density distribution and, thus, faces the option to relax to a new configuration. In case of relaxation, the dopant molecule responds with a change in the helium solvation shift which can be observed in dispersed emission. Without relaxation, the dopant exhibits an identical solvent shift in dispersed emission and in fluorescence excitation. In fact, in helium droplets phthalocyanine exhibits dual emission which fits perfectly to the model discussed. The entire emission spectrum can be split into two overlapping spectra which are identical in the vibrational frequencies and in the intensity pattern; however, they are different in the integral intensity as well as in the solvent shift. One part exhibits a solvent shift of −42 cm${}^{-1}$ and, thus, coincides in the origin with the excitation, and a second part exhibits a solvent shift of −52.8 cm${}^{-1}$. As depicted in Figure 2 the dual emission reveals a branching in the decay path of the electronically-excited dopant molecule. The branching ratio scales monotonously with the excess excitation energy as shown in panel (b) of Figure 3. Identical line widths throughout the dual emission speak for a rigid solvation layer with well localized helium atoms in both electronic states and in both configurations. The change in the solvent shift visualizes the change in the electron density distribution of the dopant molecule upon electronic excitation. The back relaxation after radiative decay to the electronic ground state has been observed in a pump-probe experiment [16]. The corresponding rate constant was determined to roughly 0.2 MHz, which is small compared to energy dissipation (10${}^{6}$ MHz). A rate constant of 0.2 MHz speaks for tunneling through a barrier separating local and global minima in the configuration potential of the dopant helium solvation complex (cf. Figure 2). Corresponding observations for Mg-phthalocyanine and for van der Waals clusters of phthalocyanine with an Ar atom confirm the model for the interpretation of dual emission [30,36]. Even though we are not yet able to provide a quantitative analysis, this particular helium-induced spectroscopic signature in the dispersed emission reveals a direct access to the electron density distribution of electronically excited molecular states.

### 2.2. Line Shape Analysis

**Figure 4:**
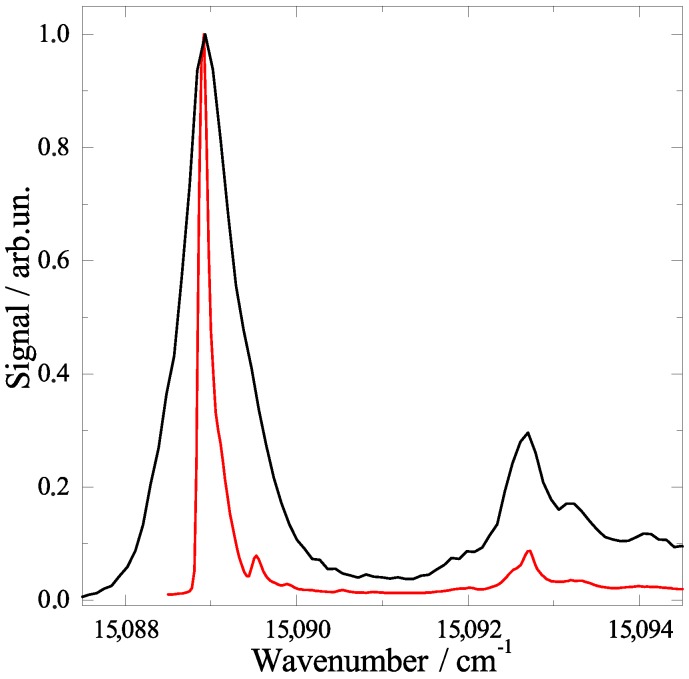
Electronic origin in the fluorescence excitation spectrum of phthalocyanine in helium droplets once beyond (black) and once below (red) the saturation limit. The leading intense peak represents the zero phonon line (ZPL) which is accompanied to the blue by the phonon wing (PW).

After the analysis of resonance frequencies, the line shape of individual resonances in these spectra will now be of interest. Under the given experimental conditions the line shapes in our dispersed emission spectra are dominated by an apparatus function and are therefore not suited for further analysis. However, for our fluorescence excitation spectra the apparatus function is negligible compared to the experimental line width. The electronic origin is unique in exhibiting an inhomogeneous line shape which is highly asymmetric as discussed below. For all of the vibronic resonances which are not perturbed by coupling to the second electronically excited state, perfect Lorentzian line shapes have been recorded [16]. The corresponding line widths (cf. Figure 3 panel (a) left ordinate) can directly be translated to the excited state decay rates (cf. Figure 3 panel (a) right ordinate) which are obviously mode-specific. The corresponding life times scatter between 3 ps and 20 ps, however, without correlation to the mode energy. Some state specific properties other than the energy are involved. Internal vibrational redistribution (IVR) might be the mechanism responsible for the excited states lifetime. The highly efficient dissipation of excess excitation energy into the helium droplet works perfectly for low energy quanta. However, a high energy quantum may reside in the dopant as shown for vibrational excitation of HF in helium droplets [44,45]. However, vibrational excess energy of electronically excited phthalocyanine was observed to fully dissipate into the helium droplets independent on the value of the energy quanta. For polyatomic molecules of the size of phthalocyanine, the energy dissipation is accomplished by first splitting into low energy quanta via IVR [46,47]. Thereby, the coupling from one quantum in a high energy mode to multiple quanta of one or several low energy modes is not expected to correlate with the mode energy. Instead, it depends on coupling constants among vibrational modes. According to this interpretation, the Lorentzian line widths in the fluorescence excitation spectrum reveal quantitative information on the intramolecular vibrational mode coupling of phthalocyanine. In contrast to the perfect Lorentzian line shapes of vibronic transitions, the electronic origin shows an asymmetric line shape which is indicative for inhomogeneous line broadening. However, it needs to be noted that the line shape is highly sensitive to the laser intensity. As shown in Figure 4 it varies from a nearly symmetric line, which is neither of Lorentzian nor of Gaussian shape (black line), to a highly asymmetric line shape including some substructure (red line). In the first case, the full power of the laser (up to 10${}^{2}$ W/cm${}^{2}$) causes severe saturation broadening. In the second case, upon attenuation of the laser by two orders of magnitude the factual physical line shape of the molecular system is resolved. As discussed in more detail in [38,39], the asymmetry in the line shape with a steep rise on the red edge and an extended tail at the blue side reflects the experimental droplet size distribution. As is well known, the red shift of the electronic transition increases monotonously with the number of attached helium atoms. It finally converges to the bulk value. With the help of the well-known droplet size distribution and the dopant to helium van der Waals parameters, the line shape could be simulated quantitatively [38,39]. Moreover, the model calculation could be inverted to deduce the parameters of the droplet size distribution from the experimentally observed line shape. Below 15,091 cm${}^{-1}$ the superimposed substructure can be assigned to ${}^{13}$C isomeric variants of phthalocyanine, while the signal beyond 15,091 cm${}^{-1}$ represents the PW [18,21,48]. According to this analysis, homogeneous contributions and rotational fine structure must be negligible within the 0.1 cm${}^{-1}$ half width of the inhomogeneous line shape at the electronic origin. According to this interpretation the asymmetry in the line shape of the electronic origin is expected to be present for all dopant species and can be resolved under the condition that the excited state lifetime is long enough. As will be shown below, there are remarkable differences even among structurally related systems. Again, the line shape analysis revealed information on both the dopant species as well as the helium environment. Most important, the Lorentzian line widths of vibronic transitions provide evidence for IVR, a process already assumed for line width studies in the gas phase [40,42]. In addition the inhomogeneous line shape at the electronic origin reveals the droplet size distribution.

Finally, the line shape at the electronic origin of phthalocyanine deserves further discussion. As mentioned above, the asymmetric line shape reflects the droplet size distribution. Slightly simplified, one can state that the larger the radius of the polarizable environment, the larger the red shift at the electronic origin. However, since the range of van der Waals forces is finite, the red shift approaches a limiting value which is identical to the red shift observed in bulk helium. Beyond a certain droplet size the dopant does not discern any more the limiting radius of the droplets. When approaching these conditions we expect vanishing inhomogeneity. In fact, for super-large droplets with on average more than 10${}^{6}$ helium atoms, the asymmetric tail to the blue vanished and a double peak structure could be resolved at the electronic origin of phthalocyanine. With appropriate modifications, namely considering a solvation complex, in a helium droplet the envelope of a nearly symmetric oblate top rotor at 0.37 K fits to the double peak feature [21]. So far, the experiment fulfills the expectations. However, concerning the convergence of the solvent shift, the experimental observation is counterintuitive. Instead of converging to a maximum value in the red shift, the solvent shift passes a maximum value for droplets of about 10${}^{5}$ helium atoms. Then the solvent shift undergoes a turnaround so that the double peak appears with a reduced red shift. Similar observations reported for tetracene in helium droplets were taken as evidence for capture of the dopant species by quantum vortices [17], a special feature of quantized rotational momenta in superfluid helium. However, this very interesting idea requires either proof by theoretical simulations or by a more systematic experimental investigations including other dopant molecules. The latter is the subject of current work in our laboratory. In particular, the spectroscopic features reflecting microsolvation in helium droplets are expected to be present with minor modifications for all dopant species. What was revealed by a detailed investigation of the inhomogeneous line shape at the electronic origin of phthalocyanine fulfills the expectations on molecules embedded into a polarizable environment finite in size. In the case of phthalocyanine, the moment of inertia of the freely rotating solvation complex could be determined. A non-monotonous development of the solvent shift in the electronic transition requires further experimental investigation. For a different, however, similar purpose, we have extended our experimental work by studying porphyrin derivatives and in addition some phthalocyanine derivatives [49]. By comparison of experimental data obtained for related molecular compounds, the empirical interpretation discussed for phthalocyanine can be cross checked and proven or falsified. In the following, fluorescence excitation spectra mainly of porphyrin derivatives will be reviewed with particular emphasis on the electronic origin.

## 3. Porphyrin Derivatives at 0.37 Kelvin inside Helium Droplets

**Figure 5:**
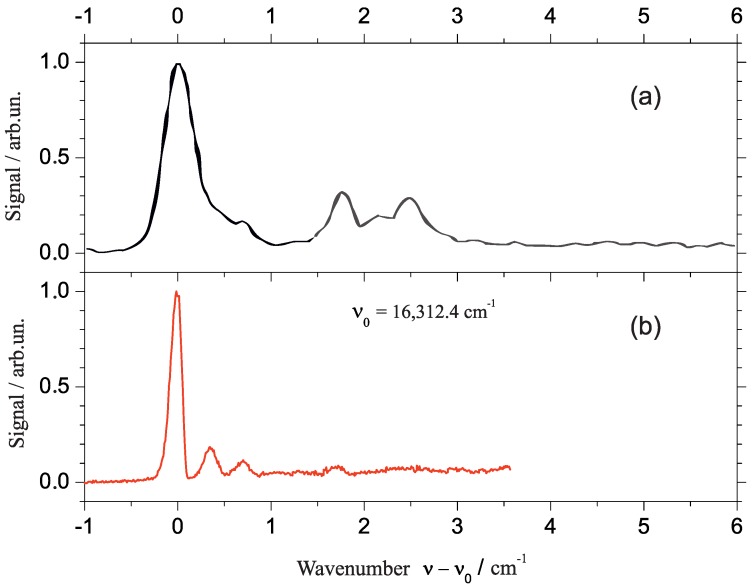
Electronic origin of the fluorescence excitation spectrum of porphyrin in helium droplets once recorded beyond (**a**) and once below (**b**) the saturation limit.

**Figure 6:**
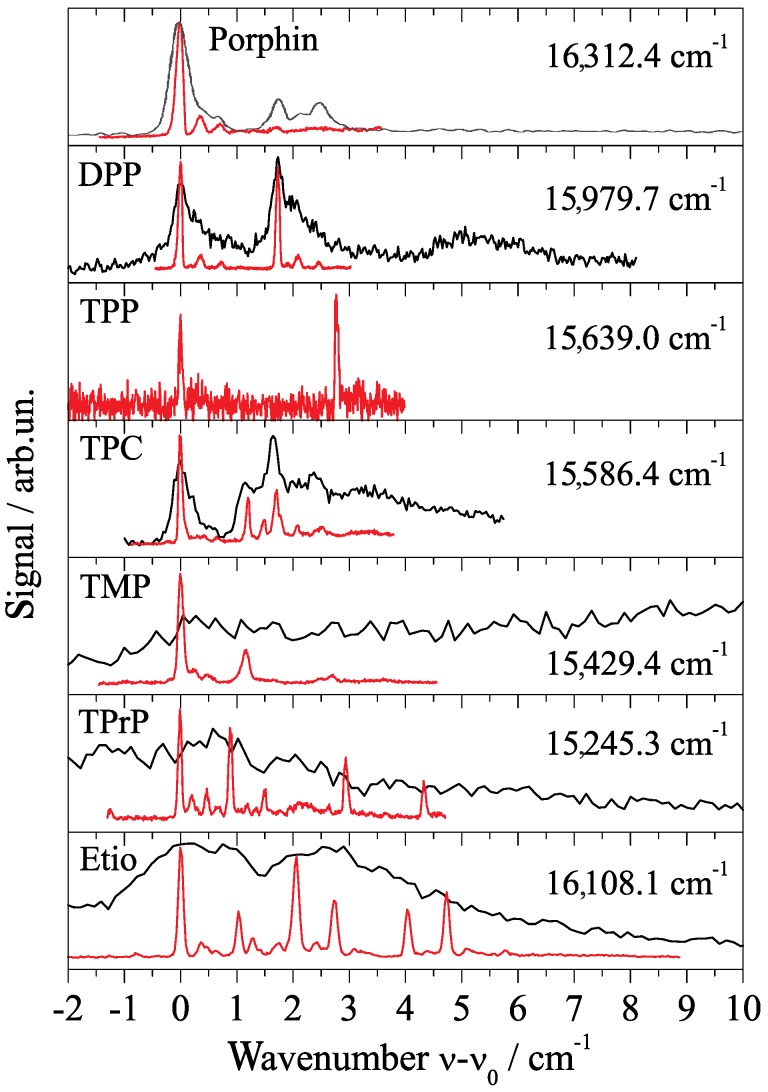
Electronic origin of the fluorescence excitation spectra of porphyrin derivatives (as indicated in each panel) in helium droplets all recorded with laser intensity beyond (black) and below (red) the saturation limit. DPP: diphenyl-porphyrin; TPP: tetraphenyl-porphyrin; TPC: tetraphenyl-chlorin; TMP: tetramethyl-porphyrin; TPrP: tetrapropyl-porphyrin; Etio: tetraethyl-tetramethyl-porphyrin. For each dopant species the absolute wavenumber ${\bar{\nu }}_{0}$ is added to the panel.

When looking at the electronic origin of porphyrin in helium droplets (cf. Figure 5) in comparison to phthalocyanine (cf. Figure 4), the sensitivity of the helium-induced fine structure on the shape of the dopant molecule becomes evident. This is further exemplified by the electronic origin of a series of porphyrin derivatives shown in Figure 6. Porphin and phthalocyanine derivatives with different substituents (mostly hydrocarbon moieties) in the periphery of the core unit are readily commercially available. The investigation of a series of differently substituted derivatives allows us to study the influence of the substituent on the vibrational fine structure of the molecule. Of particular interest is the presence or absence of low energy torsional or bending modes of the substituents. Moreover, substituents might be effective on the energy dissipation from the excited molecule into the helium droplet. Finally, it allows to study the influence of the dopant molecule on helium-induced spectral features such as the PW and the ZPL fine structure. Thereby, we learn about both intra- and intermolecular processes and in particular about microsolvation in superfluid helium. In comparison to the observations made for phthalocyanine, the influence of the droplet size distribution on the line shape at the electronic origin is an interesting issue. The fine structure assigned to ${}^{13}$C isomers and the spectral shape of the PW is another issue. This all is subject of current work in our laboratory. In the following we will restrict the discussion on the comparison of the helium-induced fine structure at the electronic origin recorded for optimized signal intensity of singly doped droplets. Besides bare porphyrin, diphenyl-porphyrin (DPP) and tetraphenyl-porphyrin (TPP) will be discussed, as well as tetramethyl-porphyrin (TMP), tetrapropyl-porphyrin (TPrP), and finally tetraethyl-tetramethyl-porphyrin (Etio). In addition, tetraphenyl-chlorin (TPC) has sneaked in as a well known byproduct of the synthesis of TPP.

The discussion starts with the electronic origin of bare porphyrin in helium droplets which is shown twice in Figure 5. In panel (a) it was recorded for high laser intensity far beyond the saturation limit and in panel (b) it was recorded below the saturation limit. As expected, saturation broadening hides much of the spectral fine structure. The single intense and asymmetric peak shown in panel (a) splits up into a leading intense and narrow resonance accompanied by two much weaker peaks shifted by about 0.4 and 0.7 cm${}^{-1}$ to the blue (cf. Figure 5b). Compared to this signal trio the oscillator strength of the signal beyond 1 cm${}^{-1}$ is greatly reduced. Therefore, it almost disappeared for low laser intensity (cf. panel (b)) and is still below saturation in panel (a). According to the different oscillator strength, the signal beyond 1 cm${}^{-1}$ was assigned to the PW of pophyrin in helium droplets while the leading trio represents the ZPL [18]. The solvent shift is significantly reduced to only 8±4 cm${}^{-1}$ [28]. The PW as well as the triple peak fine structure are helium induced. The triple peak feature of the ZPL does not fit to what is expected for ${}^{13}$C isomeric variants of porphyrin. It is also far from the rotational fine structure expected for porphyrin in helium droplets at 0.37 K. On comparison with the corresponding spectral section of phthalocyanine (Figure 4), it becomes apparent that helium-induced fine structure at the electronic origin is highly specific for the dopant molecule. Even though there is a structural similarity between porphyrin and phthalocyanine, this similarity is not reflected by the spectral shape at the corresponding electronic origin. What on the first view appears to be a discouraging experimental result turns into an advantage. The different helium-induced fine structure reveals the high sensitivity of the helium environment on the dopant species. Unfortunately, we are still far from deciphering molecular structure from these spectral features. However, increasing collections of experimental data may once help to understand and predict those spectral features.

In order to concentrate on the essentials, a summary of the electronic origin of six porphyrin derivatives measured in our laboratory is presented in Figure 6. All spectra are plotted to the same scaling in the abscissa which starts at the corresponding origin as indicated in each panel. Such comparative investigations aim to recognize particular characteristic features that might help to recognize what is common to all and what is specific for each dopant species. Since theoretical treatment of a solvation complex embedded into a quantum fluid is subject of current development, the comparative analysis of experimental data might reveal deeper insight into the phenomenon of microsolvation in superfluid helium droplets and, thus, even guide theoretical activities in this field. The top panel of Figure 6 repeats the electronic origin of porphyrin already shown in Figure 5 while the next two panels show the origin of DPP and TPP, respectively. The similarity of the triple peak feature in the spectrum of DPP (second panel) to that of porphyrin (top panel) is striking. Despite the poor signal to noise ratio obtained for the spectrum of TPP (third panel), we recognize the same triple peak feature certainly hardly overcomes the signal to noise level. In both of the phenylated porphyrin derivatives there is a doublet of the triple peak feature which is readily explained by tunneling doubling among conformational isomers with respect to the tilt angles of the phenyl moieties within these porphyrin derivatives. The frequency shift due to phenylation from bare via diphenyl- to tetraphenyl-prophyrin appears to be almost additive. As can be calculated from the numbers for ${\bar{\nu }}_{0}$ given in Figure 6 it amounts to about −168 cm${}^{-1}$ for each step. Unfortunately, corresponding spectra from gas phase samples are not available for comparison. It should be noted that resolving these spectroscopic details for porphyrin derivatives in the gas phase might be very difficult because of the low temperature conditions required. For this series of three porphyrin derivatives the triple peak feature can be identified as a characteristic motive for helium solvation, whereas the doubling of the triple peak motive for the two phenylated porphyrines is of purely intramolecular origin. Thus, only the tunneling doublet is expected to be present in the corresponding gas phase spectrum while the triple peak feature is expected to be reduced to a single peak.

The reappearance of the triple peak motive as observed in the first three spectra in Figure 6 does not continue as clearly for the remaining spectra plotted in Figure 6. The addition of substituents such as methyl, propyl, or even a mixture of methyl and ethyl moieties attached to the porphyrin core reveals significant differences in the spectral shape of the electronic origin as compared to bare porphyrin or its two phenyl-substituted derivatives. However, at a second and less tight look at least TMP and TPrP reveal a related motive consisting of a leading intense peak accompanied to the blue by at least two much weaker peaks. With a certainly slightly biased view one might state that all of the porphyrin derivatives reveal the characteristic motive of an intense leading peak followed to the blue by an additional signal which is much weaker in the intensity. Variations are found in the spectral width and in the intensity profile of this motive. Since we assume this motive is helium induced, the similarity throughout the series of dopant species might reflect the similarity in the shape of the dopant to helium interaction potential. At least the core porphyrin moiety provides similar if not identical corrugation in the interaction potential to the surrounding helium. Variations in the spectral width, the intensity profile, and the individual line widths within this motive might originate from the different substituents of the porphyrin derivatives. As known from numerous experiments, the lifetime of excited states of molecules is limited by the process of energy dissipation finally into the helium environment. Thereby, low energy quanta are more efficient for dissipation than large quanta. Thus, the substituents may act as an antenna once more and once less effective for energy dissipation. According to such rather empirical thoughts, the variations in the spectral appearance of a kind of triple peak motive are comprehensible, while the similarity among porphyrin, DPP, and TPP is remarkable. So far, the comparative investigation of the helium-induced fine structure reveals empirical insight into microsolvation in superfluid helium droplets.

Finally, the line widths recorded in the spectra have to be considered. This parameter requires experimental conditions avoiding saturation broadening. Under severe saturation the entire fine structure may vanish as shown by the black lines in Figure 6. As can also be seen in Figure 6 the line width of individual resonances (red lines) varies from dopant to dopant species altogether by a factor of about 2. The line widths in the spectra of TPrP and TPC are roughly half of the line width of bare porphyrin. The smallest line width recorded for TPC is 0.05 cm${}^{-1}$. It is definitely smaller than the unresolved rotational fine structure as measured for phthalocyanine in superfluid helium droplets consisting of more than 10${}^{6}$ helium atoms [21]. In the case of the porphyrins, even though the average droplet size was only 10${}^{4}$ atoms, the underlying droplet size distribution is of minor influence on the line shape of the ZPL. Moreover, the entire line width is smaller than the rotational fine structure observed for phthalocyanine in helium droplets. On the other hand, porphyrin exhibits a rotor symmetry identical to phthalocyanine and, therefore, is expected to exhibit almost identical rotational fine structure in helium droplets as resolved for phthalocyanine. This might raise questions in the assignment of the double peak fine structure of phthalocyanine to a rotational fine structure. By means of Stark spectroscopy we are about to get a deeper insight into contributions of the rotational fine structure to the line shape of the ZPL at the electronic origin.

## 4. Phthalocyanine Derivatives and Tetraphenyl-Chlorin at 0.37 Kelvin in Superfluid Helium Droplets

**Figure 7:**
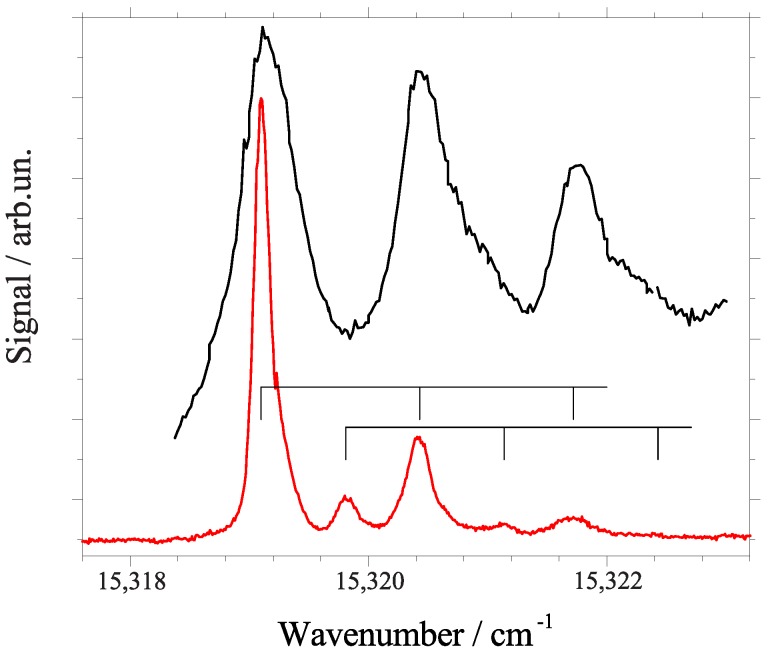
Electronic origin of the fluorescence excitation spectrum of AlCl-phthalocyanine in helium droplets recorded with laser intensity beyond (black line) and below (red line) the saturation limit. Below saturation limit, a fine structure is resolved which consists of two triple peak features as indicated by two combs. For details see text.

At the end we would like to highlight two particular spectroscopic details which emphasize the problem of correct interpretation of spectroscopic data. The first concerns porphyrin and its phenylated derivatives (cf. top three panels of Figure 6). With all restrictions discussed above, the triple peak motive of porphyrin is present for both DPP and TPP. However, the simple addition of one hydrogen atom which causes the change from TPP to TPC is accompanied by severe changes in the fine structure at the electronic origin (cf. 4th panel from top in Figure 6). However, this additional atom has severe impact on the electron density distribution. The minor spectroscopic response to phenylation contrasts to the strange changes upon simple addition of one H atom. Obviously, the electron density distribution is a major factor for helium-induced spectral features obtained for the ZPL and the PW at the electronic origin of a dopant species. Secondly, the electronic origin of a phthalocyanine derivative, namely of AlCl-phthalocyanine is presented in Figure 7. It is shown twice, once beyond (black) and once below (red) the saturation limit. It is an additional example for the loss of information due to saturation. Certainly, the exchange of the two inner hydrogen atoms by an aluminum atom almost at the center of mass and an additional Cl atom reaching out of the phthalocyanine plane is a strong variation concerning the molecular symmetry as well as the electron density distribution. Thereby, the inversion symmetry gets lost, a permanent electric dipole moment is present, and additional degeneracies are present due to the C${}_{4}$ symmetry axis. Therefore, the difference in the spectral fine structure at the electronic origin of TPC and of phthalocyanine is no surprise. Rather surprisingly is the similarity to the triple peak feature recorded for porphyrin and its phenylated derivatives. The assignment of two triple peak features as indicated by the combs in Figure 7 are justified by dispersed emission spectra as outlined in [50]. These two examples are typical for the interpretation of helium–induced features in electronic spectra of molecules in helium droplets. A theoretical treatment of the experimental data discussed in this review has to deal with a dopant helium solvation complex which in the case of phthalocyanine consists of up to 50 particles [20]. Moreover, in addition to the electronic ground state one of the electronically excited states has to be considered. Finally, the interaction of the solvation complex with the surrounding helium droplet has to be considered. Much work is still needed in order to obtain quantitative simulation of the helium induced fine structure.

Finally, we would like to add a note on electronic spectroscopy of phthalocyanine derivatives such as AlCl-phthalocyanine, GaCl-phthalocyanine, and AlOH-phthalocyanine. All three of these polar pthalocyanine derivatives have been investigated by means of electronic spectroscopy in a homogeneous Stark field [51,52]. Even though these experiments have been performed at significantly reduced spectral resolution, detailed insight into the orientation of the permanent dipole moment and the transition dipole moment within the dopant species was obtained. For GaCl-phthalocyanine the fashionable technique of diffraction imaging has been applied. In contrast to X-ray diffraction imaging experiments, this project dealt with electron diffraction in a table top experimental setup. From the diffraction image the average number of helium atoms attached to the dopant species could be deduced [53]. So far, these experimental approaches are still in development. However, both will provide additional insight into the details of both the dopant species and the helium environment.

## 5. Summary

This article reviews a very special approach to phthalocyanine, porphyrine, and some of the corresponding derivatives, namely, electronic spectroscopy of these compounds when doped into superfluid helium droplets. The unique properties of superfluid helium droplets such as low temperature and vanishing viscosity allow for brilliant spectral resolution. Thus, many of the properties of this class of molecules could be confirmed by the combined investigation of fluorescence excitation and dispersed emission spectra in helium droplets.

Part of the helium-induced spectral features reveals dopant properties which cannot be observed in the gas phase. In the first place it was the doubling observed in the emission spectrum of phthalocyanine which revealed dynamics initiated by the change of the electron density distribution. In general, helium-induced spectral structures are often a response to the change of the electron density distribution. The helium environment provides an enhanced sensitivity to these processes.

The other important aspect of electronic spectroscopy in helium droplets is microsolvation of molecules in superfluid helium. From the purely physical point of view those experiments provide access to one of the weakest van der Waals interaction and, thus, to the bottom of what might be relevant for intermolecular interaction. Even though we have discussed one particular family of molecular compounds, namely, phthalocyanine, porphyrin and some of their derivatives, the variation in the helium-induced fine structure in the ZPL and the spectral shape of the PW is remarkable and reflects the high sensitivity of the experiment to structural changes of the molecular species under investigation.

Much work is still to be done in order to transform the spectral fine structure into the structure of the dopant molecule. However, the present experimental findings provide encouragment to accept this challenge.
